# Dual-Energy Contrast-Enhanced Spectral Mammography: Enhancement Analysis on BI-RADS 4 Non-Mass Microcalcifications in Screened Women

**DOI:** 10.1371/journal.pone.0162740

**Published:** 2016-09-09

**Authors:** Yun-Chung Cheung, Yu-Hsiang Juan, Yu-Ching Lin, Yung-Feng Lo, Hsiu-Pei Tsai, Shir-Hwa Ueng, Shin-Cheh Chen

**Affiliations:** 1 Department of Medical Imaging and Intervention, Chang Gung Memorial Hospital at Linkou, Taoyuan, Taiwan; 2 Department of Surgery, Chang Gung Memorial Hospital at Linkou, Taoyuan, Taiwan; 3 Department of Pathology, Chang Gung Memorial Hospital at Linkou, Taoyuan, Taiwan; 4 Medical College of Chang Gung University, 5 Fuxing Street, Guishan Township, Taoyuan County, Taoyuan, Taiwan; Fu Jen Catholic University, TAIWAN

## Abstract

**Background:**

Mammography screening is a cost-efficient modality with high sensitivity for detecting impalpable cancer with microcalcifications, and results in reduced mortality rates. However, the probability of finding microcalcifications without associated cancerous masses varies. We retrospectively evaluated the diagnosis and cancer probability of the non-mass screened microcalcifications by dual-energy contrast-enhanced spectral mammography (DE-CESM).

**Patients and Methods:**

With ethical approval from our hospital, we enrolled the cases of DE-CESM for analysis under the following inclusion criteria: (1) referrals due to screened BI-RADS 4 microcalcifications; (2) having DE-CESM prior to stereotactic biopsy; (3) no associated mass found by sonography and physical examination; and (4) pathology-based diagnosis using stereotactic vacuum-assisted breast biopsy. We analyzed the added value of post-contrast enhancement on DE-CESM.

**Results:**

A total of 94 biopsed lesions were available for analysis in our 87 women, yielding 27 cancers [19 ductal carcinoma in situ (DCIS), and 8 invasive ductal carcinoma (IDC)], 32 pre-malignant and 35 benign lesions. Of these 94 lesions, 33 showed associated enhancement in DE-CESM while the other 61 did not. All 8 IDC (100%) and 16 of 19 DCIS (84.21%) showed enhancement, but the other 3 DCIS (15.79%) did not. Overall sensitivity, specificity, positive predictive value, negative predictive value and accuracy were 88.89%, 86.56%, 72.72%, 95.08% and 87.24%, respectively. The performances of DE-CESM on both amorphous and pleomorphic microcalcifications were satisfactory (AUC 0.8 and 0.92, respectively). The pleomorphous microcalcifications with enhancement showed higher positive predictive value (90.00% vs 46.15%, p = 0.013) and higher cancer probability than the amorphous microcalcifications (46.3% VS 15.1%). The Odds Ratio was 4.85 (95% CI: 1.84–12.82).

**Conclusion:**

DE-CESM might provide added value in assessing the non-mass screened breast microcalcification, with enhancement favorable to the diagnosis of cancers or lack of enhancement virtually diagnostic for non-malignant lesions or noninvasive subgroup cancers.

## Introduction

Despite the influence of breast tissue density on breast cancer detection, mammography remains an important breast imaging technique [1, 2]. Mammography is a cost-effective method and particularly beneficial in detecting suspicious malignant microcalcifications. About 20–25% of the American College of Radiology breast imaging reporting and data system (ACR BI-RADS), category 4 microcalcifications without an associated mass (suspicious abnormality where a biopsy is recommended) were subsequently shown to be malignant [3–5]. Unfortunately, their morphologic appearance of ACR BI-RADS 4 suspicions on mammograms was variable with the probability of cancer ranging from 2–95%. Although screening mammography was shown to reduce mortality from breast cancer, whether or not it results in overdiagnosis and unnecessary biopsies is still a matter of persistent debate [6]. This question has provoked clinical and research interest to find a new diagnostic approach to better characterize suspicious microcalcifications without an associated mass in asymptomatic patients.

Recently, a technical revolution in digital imaging has facilitated the development of advanced mammographic imaging including tomosynthesis and dual-energy contrast enhanced spectral mammography (DE-CESM). Tomosynthesis can potentially resolve superimposed images of cancer from fibroglandular breast tissues on conventional mammography, while DE-CESM can enhance the detection of occult cancer due to tumor angiogenesis. Both methods have been shown to improve cancer detection [7–11]. In addition to the morphology and distribution of microcalcifications seen using conventional mammography, DE-CESM also displays cancer with alterative information because of iodine uptake. The presence of iodine uptake highlights possible underlying pathologies. However, the additional benefit of showing different types of suspicious microcalcifications is still unclear.

To the best of our knowledge, a preliminary analysis of DE-CESM on the women with suspicious microcalcifications had been previously reported [12]. Here, we further investigated the role of enhancement of DE-CESM in evaluating the suspicious malignant microcalcifications without associate mass in screened women that might be warranted in this research area.

## Patients and Methods

### Patient Selection

To assess the performance of DE-CESM in diagnosing microcalcifications in screened women, we retrospectively reviewed all patients receiving DE-CESM from February/2012 to June/2015. With the approval of our institution’s review board (Chang Gung Medical Foundation: Number104-8759B) and all patients gave their signed agreements to participate, all patients enrolled in this study were selected based on the following criteria: (1) had an ACR BI-RADS-4 microcalcification referral from the National Screening Program of our country; (2) had no related mass after clinical assessment consisting of a physical examination and breast sonography; (3) had a pathological diagnosis of microcalcifications using stereotactic-guided vacuum-assisted biopsies; and (4) received DE-CESM before being biopsied. We chose only patients who received mammographic stereotactic core needle biopsy because the suspected microcalcification or parenchymal enhancement can be easily targeted under mammographic biopsy, which could further minimized potential errors in tissue sampling during surgery.

DE-CESM was not compulsory prior to performing the stereotactic breast biopsy. We explained the advantages and risks of undergoing additional examination with DE-CESM to all patients, and all patients gave their signed agreements to participate.

Exclusion criteria that prohibited patients from undergoing a DE-CESM examination included: 1) contraindications of renal functional impairment (serum creatinine >1.0 mg/dL and a glomerular filtration rate <60 mL/min/1.73 m^2^); 2) pregnancy; 3) lactation; 4) a history of an allergic reaction to contrast medium; and 5) certain systemic diseases such as hyperthyroidism.

### DE-CESM Protocol

DE-CESM was performed using a commercial mammography apparatus (Senographe Essential CESM; GE Healthcare, Buc, France) that provided intermittent exposure to low and high energy in 1–2 s intervals during a single breast-compressed position. Using molybdenum or rhodium with automatic filter selection, alternative exposures achieved the necessary acquisition of the X-ray spectrum below and above the k-edge of iodine (33.2 keV) for successful image subtraction recombination. A subtracted image was then created after masking the different attenuations on the low- and high-energy images and eliminating the noise of non-enhanced anatomical structures. The residual net attenuation indicated the enhancement secondary to the presence of iodine uptake. This technique allowed us to assess any enhancement that may have correlated suspicious, subtle microcalcifications on CESM with conventional mammograms at approximately the same time and position between images.

Consecutive mammogram acquisitions were sequentially performed with craniocaudal (CC) and mediolateral oblique views of the bilateral breasts within 2–6 min after the start of a single-bolus injection of non-ionic contrast medium (Omnipaque 350 mg I/mL; GE Healthcare, Dublin, Ireland) at a rate of 3 mL/s for a total dose of 1.5 mL/kg body weight via an intravenous catheter that was inserted into the forearm prior to the examination. A nurse and mammographer monitored extravasation or allergic reaction to the contrast medium throughout the procedure. Patients were requested to hold their breath during mammographic exposure to avoid motion artifacts. Low- and high-energy acquisitions were immediately captured digitally and recombined yielding a subtracted mammogram, such that conventional low-energy mammography and CESM images were obtained in each single-study view. Eight mammography images from bilateral breasts were obtained per examination.

### Stereotactic Biopsy Procedure

Stereotactic core needle breast biopsies using the same mammographic machine with an add-on biopsy unit (Senographe Essential CESM; GE Healthcare) were subsequently performed using 10-gauge vacuum-assisted needles (Bard Vacora, Covington, CA, USA) on the target microcalcifications. Microcalcifications with associated enhancement were first identified and were the first priority for the biopsy. When there was no associated enhancement, the biopsy was performed on the most suspicious microcalcifications based on their morphology. Finally, specimen radiography was routinely used to conduct sufficient sampling over the microcalcifications; and specimens with or without calcifications were identified and separately submitted for pathologic evaluation. Breast pathology specialists were responsible for the histopathologic diagnosis.

### Data Collection and Analyses

To assess the diagnostic feasibility of using enhancement in DE-CESM, the presence of enhancement over the site of microcalcifications was categorized as positive for malignancy, and its absence for noncancerous lesions. With reference to the histological diagnosis, the true positive, true negative, false positive, false negative, sensitivity, specificity, positive predictive value (PPV), negative predictive value (NPV) and accuracy for diagnosing malignancies were determined.

The biopsied microcalcifications were classified as amorphous or pleomorphic according to mammographic morphologies. Diagnostic results were individually counted for all types of microcalcifications, amorphous and pleomorphic, as well as analyzed for their performance using receiver operating characteristic (ROC) curves according to the Hanley and McNeil formula, and cancer probabilities were compared using the odds ratios.

## Results

A total of 94 consecutive biopsied microcalcifications from 87 women (45–66 years old, average 54 years old) who fulfilled the inclusion criteria were enrolled for analysis. All of the enrolled patients were Asians (Taiwanese) with no previous known history of breast or gynecological cancer. These patients were referred for both CESM and stereotactic biopsy in our tertiary medical center due to ACR BI-RADS-4 suspicious microcalcifications from screening mammography as supported by the National Screening Program of our country. Seven patients were biopsied at two different sites in the same session using the stereotactic biopsy procedure. Twenty-seven (28.72%) suspicious microcalcifications were pathologically diagnosed as cancer [19 ductal carcinoma *in situ* (DCIS), and 8 invasive ductal cancer (IDC)], 32 (34.04%) as atypia [10 atypical ductal hyperplasia (ADH) and 22 flat epithelial atypia (FEA)], and 35 (37.23%) as benign breast pathologies (eight nonspecific benign calcifications, six adenomas, five proliferative diseases, five nonproliferative diseases, five nonspecific hyperplasias, three fibroadenomas and three fibrocystic diseases). Pathologic diagnoses are listed in Table 1. The diagnosed cancers or atypia were recommended for surgery and the diagnosed benign lesions for interval follow up.

**Table 1:** Enhancement from DE-CESM in different histologic diagnoses.

Histological Diagnosis	Enhanced (%)	Unenhanced (%)
**Malignant (27)**		
IDC (8)	8 (100)	0 (0)
DCIS (19)	16 (84.21)	3 (15.79)
**High Risk Lesions (32)**		
ADH (10)	5 (50)	5 (50)
FEA (22)	1 (4.54)	21 (95.46)
**Benign Lesions (35)**		
Nonspecific Calcifications (8)	1 (12.5)	7 (87.5)
Adenosis (6)	2 (33.33)	4 (66.67)
Proliferative (5)	0 (0)	5 (100)
Non-Proliferative (5)	0 (0)	5 (100)
Nonspecific Hyperplasia (5)	0 (0)	5 (100)
Fibrocystic (3)	0 (0)	3 (100)
Fibroadenoma (3)	0 (0)	3 (100)

Abbreviations: DE-CESM (dual-energy contrast enhanced subtracted mammography), IDC (invasive ductal carcinoma), DCIS (ductal carcinoma *in situ*), ADH (atypical ductal hyperplasia), FEA (flat epithelial atypia)

### All Types of Microcalcifications

Of these 94 lesions, 33 consisting of 24 breast cancers (16 DCIS and eight IDC), six atypia lesions (five ADH and one FEA) and three benign lesions (two adenomas and one nonspecific calcification) showed associated enhancement in DE-CESM while the other 61 did not. All 8 IDC (100%) and 16 of 19 DCIS (84.21%) revealed enhancement. Otherwise, 61 microcalcifications did not enhance, including three DCIS, 26 atypical lesions and 32 benign lesions. Histological diagnoses of enhanced and non-enhanced microcalcifications are listed in Table 1.

When considering all types of calcifications, sensitivity, specificity, PPV, NPV and accuracy were 88.89%, 86.56%, 77.42%, 95.08% and 87.24%, respectively. False positive and false negative rates were 27.27% and 4.84%, respectively. Regarding to the Surveillance, Epidemiology, and End Results (SEER) Program data (National Cancer Institute, Bethesda, MD, USA), the cancer probability of having enhancement was 28.7%.

### Pleomorphic and Amorphous Microcalcifications

The morphologies of microcalcifications were composed of 41 pleomorphic and 53 amorphous microcalcifications. Pleomorphic microcalcifications were diagnosed as cancerous in 19 and noncancerous in 22 patients, while amorphous microcalcifications were found in 8 cancerous and 45 noncancerous lesions. Sensitivity, specificity, PPV, NPV and accuracy of enhancement for diagnosing pleomorphic and amorphous microcalcifications were 94.73% vs. 73%, 90.9% vs. 84.44%, 90% vs. 46.15%, 95.23% vs. 95% and 92.68% vs. 83%, respectively. The false positive rate was 10% for pleomorphic and 53.84% for amorphous microcalcifications, while the false negative rate was 4.54% and 5%, respectively. Pleomorphic microcalcifications had significantly higher positive predictive value as compared to amorphous microcalcifications (90.00% vs 46.15%, p = 0.013). The results are summarized in Table 2.

**Table 2:** Diagnostic performance of DE-CESM on different types of microcalcifications.

Populations	Overall cases	Amorphous microcalcifications	Pleomorphic microcalcifications	P value
No. of lesions	94	53	41	
Sensitivity	88.89%	75.00%	94.74%	0.201
Specificity	86.56%	84.44%	90.90%	0.707
PPV	72.72%	46.15%	90.00%	0.013
NPV	95.08%	95.00%	95.24%	1.000
Accuracy	87.24%	83.02%	92.68%	0.164

**Fig 1:**
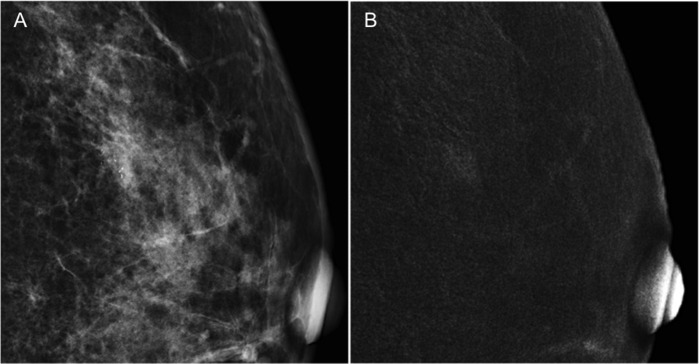
A 57-year-old woman referred from a local hospital due to suspicious malignant microcalcifications (BI-RADS 4) on biennial mammographic screening. (A) The low energy conventional mammogram on craniocaudal view showed a cluster of pleomorphic microcalcifications in the lower outer quadrant of left breast; however the sonographic evaluation revealed negative of associate mass. (B) CESM revealed a 0.7-cm irregular nodular enhancement over the associated microcalcifications. Stereotactic core needle biopsy diagnosed it to carcinoma in situ, however surgery subsequently proved it to be invasive ductal cancer.

**Fig 2:**
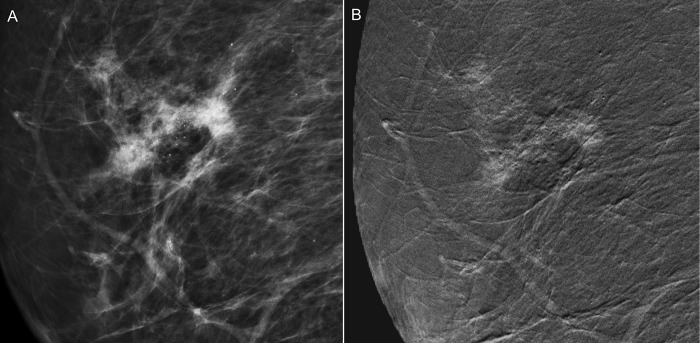
A 55-year-old woman receiving biennial mammographic screening with the finding of benign microcalcifications for 8 years was upgraded from BI-RADS category 3 to 4 in a recent examination because of increased microcalcifications. (A) Low energy conventional mammogram on mediolateral oblique view showed segmental amorphous microcalcifications in the right breast; however the sonographic evaluation did not find any associated lesion. (B) CESM revealed a 3.3-cm irregularly shaped and outlined regional enhancement associated with the area of microcalcification. Subsequently, stereotactic core needle biopsy and surgery proved it to be an invasive ductal carcinoma.

**Fig 3:**
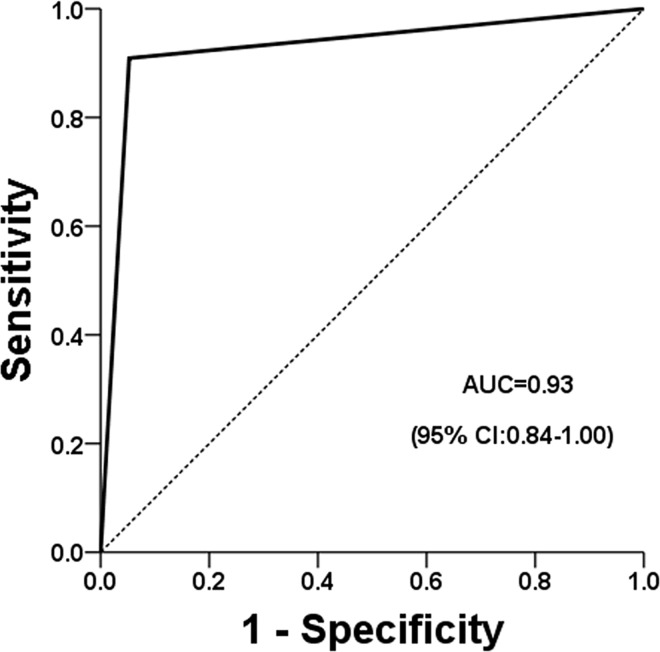
Performance of pleomorphic microcalcifications using ROC analysis.

**Fig 4:**
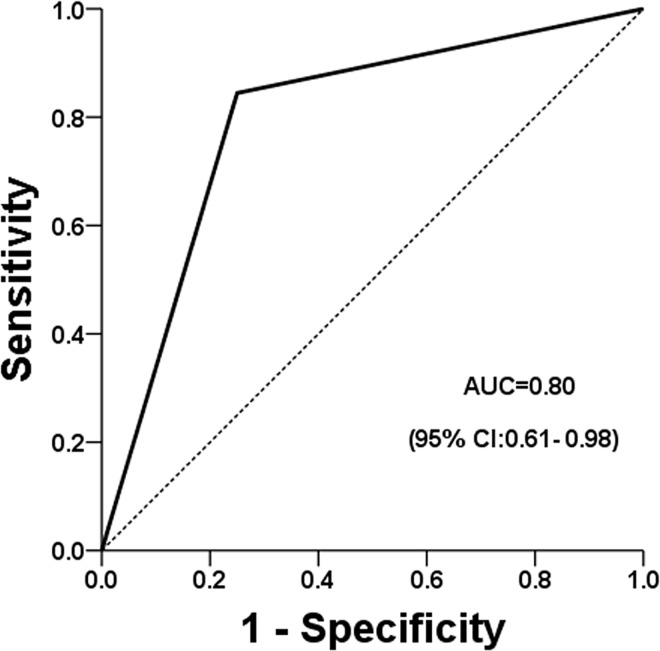
Performance of amorphous microcalcifications using ROC analysis.

The cancer probability was 46.3% for pleomorphic microcalcifications with enhancement (Fig 1) and 15.1% for amorphous microcalcifications (Fig 2); and the odds ratio was calculated to 4.85 (95% CI = 1.84–12.82). The performance, as independently determined using ROC analysis, for pleomorphic microcalcifications and for amorphous microcalcifications were both satisfactory good, with areas under the curves of 0.92 (Fig 3) and 0.8 (Fig 4), respectively.

## Discussion

### Outline of Screening and Management

A nationwide population-based biennial screening mammography program for women 50–69 years old was implemented from 2004–2009 and co-organized by the Bureau of Health Promotion and National Cancer Registry of our country, which was modified in 2009 to include women 45–49 years old. Screening results were reported according to the ACR BI-RADS standard. Patients suspected of having breast cancer (categories 0, 3, 4 or 5) were referred to the outpatient breast cancer unit for subsequent clinical and sonographic evaluation. Suspicious opaque lesions detected with sonography were subjected to real-time ultrasound-guided core needle biopsy to obtain a definitive diagnosis. For suspicious microcalcifications without an associated mass, a stereotactic-guided vacuum-assisted core needle biopsy was often recommended according to the referral policy of our hospital. Therefore, our study only included confirmed BI-RADS 4 pleomorphic or amorphous microcalcifications without an associated mass, which were essentially recommended for biopsy after assessment. However, suspicious, benign or low-risk microcalcifications were predominantly managed using interval follow-up.

Breast calcifications are common mammographic or clinical examination findings, and indeterminate microcalcifications may be those of greatest concern. Indeterminate microcalcifications can be associated with malignant, high-risk lesions or benign lesions, and thus require further imaging or tissue proof. Although the morphology and distribution of microcalcifications can help predict malignancy in some cases, the wide range of possible diagnoses creates a dilemma in clinical practice. Biopsies are usually recommended for BI-RADS 4 lesions, which may lead to excessive biopsies that may not be required if more optimal diagnostic modalities were implemented. Furthermore, most screening participants with suspicious lesions preferred to have additional information to decide whether interval follow-up or immediate biopsy was necessary.

### Conventional Mammography and Sonography on Non-Mass Microcalcifications

Detecting a suspicious calcification is not necessarily indicative of cancer. Mammography is superior to sonography for detecting microcalcifications. Morphology of microcalcifications alone is a predictive factor for cancer risk, with a reported malignancy rate of 7% for coarse heterogeneous, 11% for punctate, 20–26% for amorphous, 25–41% for fine pleomorphic and >80% for linear/branching/casting calcifications [13]. However, the benefits of higher sensitivity in detecting malignancy in suspicious microcalcifications may be countered by the disadvantage of reduced specificity. Breast sonography has limited value for detecting non-mass suspicious microcalcifications. It has been reported in the literature that only 35.3% of mammographic microcalcifications (either with a related mass or not) and 23% of cases of microcalcifications without other mammographic abnormalities could be observed using sonography [14, 15].

### Diagnostic Performance of DE-CESM

DE-CESM is an advanced technique of breast imaging that was approved by the Food and Drug Administration (FDA) of the United States for clinical use at the end of 2011. This examination protocol was implemented in our institution for clinical practice beginning in 2012. DE-CESM was found to be superior to conventional mammography because of the additional information of enhancement, as well as for preserving the high resolution of conventional mammography in the same session [9–11]. However, the actual benefits from CESM compared with conventional mammography are still uncertain in our study subpopulation.

In the clinical application of DE-CESM, determining the malignant risk of lesion enhancement may be influenced by the aggressiveness or degree of angiogenesis of the suspicious lesion. With such knowledge, the decision to recommend biopsy could be more effectively communicated to the patient.

### Tomosynthesis and Magnetic Resonance Imaging (MRI)

Advanced diagnostic modalities such as tomosynthesis and enhanced MRI of the breast have been used in evaluating suspicious microcalcifications. Digital breast tomosynthesis provides mammography-based images, which can provide further evaluation of a suspicious mass, parenchymal distortion or focal asymmetric density by solving the issue of tissue overlapping. However, detection of microcalcifications in thin slices is controversial compared with conventional mammography [7, 8, 16]. The overall sensitivity for detecting suspicious microcalcifications was higher for full-field digital mammography compared with digital breast tomosynthesis (84% vs. 75%, respectively); however there were no significant differences in overall diagnostic performance in a previous study [16]. To our knowledge, there was no prior literature showing the actual benefits of breast tomosynthesis in predicting malignancy compared with conventional mammography.

Contrast-enhanced breast MRI has been documented as having high sensitivity for breast cancer detection, ranging from 79–98% [17, 18]. One study reported that the detection rate of known cancers using DE-CESM was similar to that of enhanced MRI, with significantly improved specificity and fewer false positives [19]. A multicenter analysis of enhanced MRI to detect breast microcalcifications revealed a reasonable diagnostic performance with 87% sensitivity, 68% specificity, 84% PPV, 71% NPV and 80% accuracy [20]. Compared with our results, DE-CESM showed similar sensitivity and PPV, but had improved specificity, NPV and accuracy. Recently, the technique of applying dual energy exposure within a short time interval has allowed capture of two different energy mammograms, minimizing the problem of temporal resolution as well as restoring the spatial resolution of microcalcifications. Both conventional and contrast-enhanced subtracted mammograms can be obtained in the same session of breast positioning, which allows easy comparison of the enhanced locations to the microcalcifications. This correlation can be easily performed compared with some other methods, such as MRI.

### DE-CESM for Women Referred from Screening

A previous report regarding DE-CESM on women referred from a screening program because of a suspicious mass, abnormal density, parenchymal distortion or microcalcification showed an overall sensitivity of 100%, specificity of 87.7%, PPV of 76.2% and NPV of 100%, based on either biopsy or standard reference procedures [21]. Our study focused on screened participants referred for CESM with specific focus on the added value of enhancement to the findings of only suspicious microcalcifications; and the results were compared with the gold standard of histopathologic diagnoses by stereotactic-guided vacuum-assisted core needle biopsy directly on the suspicious microcalcifications. The results of the prior study and our study on referral-screened participants showed similarly high diagnostic performance.

From our results, we found that enhancement significantly improved the cancer prediction rate with a PPV of 46.15% for amorphous and 90% for pleomorphic microcalcifications, compared with 20% for amorphous and 28% for pleomorphic microcalcifications alone in a previous report [22]. Furthermore, all three types of microcalcifications (overall, amorphous and pleomorphic) demonstrated a high NPV of about 95%, further supporting the enhancement of DE-CESM as an adjuvant tool for assessing the screened microcalcifications.

## Limitations

Our study has several limitations. First, this study had a small number of cases. DE-CESM was initially set up at our hospital in 2012. Although we perform over 3,000 screening examinations per year, the cases of microcalcifications requiring DE-CESM was limited because most participants with low-concern microcalcifications preferred to undergo follow-up. Second, only amorphous and pleomorphic microcalcifications were analyzed in this study, because of the two most concern microcalcifications frequently discovered by screening mammography. Third, not all cases had subsequent operations, but this was also true for benign or high risk microcalcifications without associate mass in the guideline of our clinical practice.

However, the preliminary findings of DE-CESM might provide further information to help clinicians decide the most appropriate immediate action to take for suspicious microcalcifications. We hope that this study will encourage additional studies focusing on the benefits of DE-CESM in detecting cancers from different subpopulations, as well as to minimize the overtreatment of biopsy.

## Conclusion

DE-CESM might provide added value in assessing the non-mass screened breast microcalcification, with enhancement favorable to the diagnosis of cancer or lack of enhancement virtually diagnostic for non-malignant lesions or noninvasive subgroup cancers; that might facilitate the appropriate decision.

## Abbreviations

ACR: American College of Radiology
BI-RADS: Breast imaging reporting and data system
DE-CESM: Dual-energy contrast-enhanced spectral mammography
CESM: Contrast-enhanced subtracted mammography
PPV: Positive predictive value
NPV: Negative predictive value
ROC: Receiver operating characteristic
DCIS: Ductal carcinoma in situ
IDC: Invasive ductal carcinoma
ADH: Atypical ductal hyperplasia
FEA: Flat epithelial atypia
MRI: Magnetic resonance imaging
